# Using the ‘huddle’ to enhance interprofessional teamwork among nursing students through a podcast: a qualitative and exploratory pilot study

**DOI:** 10.1186/s12912-021-00747-4

**Published:** 2021-11-22

**Authors:** Ingunn Aase, Ingrid Tjoflåt, Kristin Hjorthaug Urstad

**Affiliations:** 1 Faculty of Health Sciences, University of Stavanger, Kjell Arholms Gate 41, 4036 Stavanger, Norway; 2 SHARE- Centre for Resilience in Healthcare, Faculty of Health Sciences, University of Stavanger, Kjell Arholms Gate 41, 4036 Stavanger, Norway

**Keywords:** Huddle, Podcast, Interprofessional teamwork training, Nursing education

## Abstract

**Background:**

Interprofessional teamwork is crucial for fostering healthcare performance and for minimizing adverse events. The daily huddle is an important arena for interprofessional interaction and communication between nurses and physicians in hospitals.

Although prevalence strongly rooted in clinical practice, the huddle does not seem to be a prioritized area in nursing education programs. Taking part in a huddle is traditionally something nursing students learn in their clinical studies.

Therefore, there is need for learning tools that can provide nursing students with quality assured training that can improve their preparation for interprofessional teamwork and strengthen the link between the educational institution and the field of practice. In this study, we have developed and tested a podcast to increase nursing students’ competence in interprofessional teamwork when participating in huddles.

The aim of the pilot study was to explore nursing students’ experiences with utilizing a huddle-focused podcast as a learning tool during their clinical practice studies in the hospital.

**Method:**

This qualitative and exploratory pilot study used focus group interviews.

Eleven third-year nursing students who had listened to the podcast during their practical studies at a medical hospital ward were included. The interviews were subjected to content analysis.

**Result:**

The analysis identified four categories that resonated across all participants in the focus group interviews: 1. understanding one’s own role in the huddle; 2. being encouraged to speak up; 3. using the huddle as a flexible learning tool; and 4. being authentic but not always realistic.

**Conclusion:**

Findings indicate that the huddle-focused podcast seems to be valuable for nursing students learning about interprofessional teamwork. The podcast seemed especially useful in helping the students to understand their own role and to speak up in the huddle meetings. The positive experiences with the flexibility of the podcast learning tool are promising for use in other educational settings.

## Background

To be a skilled interprofessional team worker is an important learning outcome in nurse education programs [1]. Interprofessional teamwork is crucial for fostering effective healthcare performance and for minimizing adverse medical events e.g.,[1,2,3,4,5]. The risk of poor interprofessional teamwork is linked to communication [6], and research shows that communication errors are consistently identified as the cause of unwanted incidents in health services [7, 8]. In the worst-case scenarios, this can lead to weakened patient safety and “silent kills” [9, 10].

Communication errors might have different explanations, but concepts such as hierarchy, roles, and stereotypes might form the basis for understanding communication problems [7, 8, 11, 12]. One concern is the assumption that nurses hold vital information that for various reasons is not articulated so information is not relayed to interprofessional teams. Therefore, in interprofessional teamwork it is crucial for nursing students to be proficient in clear and supportive communication when entering the practical field.

An important arena for interprofessional communication is the huddle [4]. In Norwegian hospitals, the “daily huddle” is an arena for daily interaction and communication between nurses and physicians. There is no clear standard definition of a “huddle,” but it can be described as short, face-to-face meeting held multiple times a day. It provides a look back at the previous day’s work and an overview of the patients scheduled for the day ahead (Agency of Health Care Research and Quality (AHRQ) [13]. The huddle includes discussions about managing daily patient demands and workflow, addresses patients’ special needs and preferences, and improves the provision of preventive services [14]. The huddle is the basis for a productive meeting with the patient [15, 16].

Although its prevalence is deeply rooted in clinical practice, the huddle does not seem to be a priority in nursing education programs. Our search for research revealed a lack of guidance of advice of health professionals’ roles in the huddle. A study from a medical center in the United States tested a huddle-coaching program that focused on structuring the huddle using scheduling and checklists. Findings suggested that the program was highly valued among participants and supported the clinic’s mission to deliver team-based, patient-aligned care [17].

In Norwegian nursing programs, the huddle is traditionally something students learn about through observations in the hospital ward during their clinical placement studies. Culture, incorporated routines, and experiences, all of which differ from department to department, may influence nurses’ role during the huddle and affect what is taught to the students. For this reason, it has raised concerns that learning about teamwork through huddles can become inconsistent and overly personalized [15, 18]. This is supported by findings from an integrated review of interprofessional communication in healthcare [19] which indicated a need for “handover tools” in nursing and medical education. Therefore, developing tools to prepare nursing students for interprofessional teamwork in clinical practice seems essential.

Podcasts are increasingly used in educational settings [20]. A recent integrative review of 26 studies about podcasting in nursing and midwifery education stated that the podcasts were regarded as a positive learning tool that contributed to the acquisition of new knowledge and skills, and to an improvement in clinical confidence [20]. Advantages of using podcast content for student learning have been reported to be flexibility and the ability to listen while driving, walking or engaging in other activities [21]. The review concluded that more research is needed on the use of podcasts for improving learning outcomes [20]. As only two of the studies included a qualitative explorative design, we see the need for exploring in-depth experiences of utilizing such tools for learning [22, 23].

The purpose of the pilot study was to explore nursing students’ experiences with utilizing an educational huddle-focused podcast during their clinical practice studies in the hospital setting. By broadening knowledge in this area, our goal is to develop podcasts that provides students with high-quality education in interprofessional learning.

## Methods

### Design

**Fig. 1:**
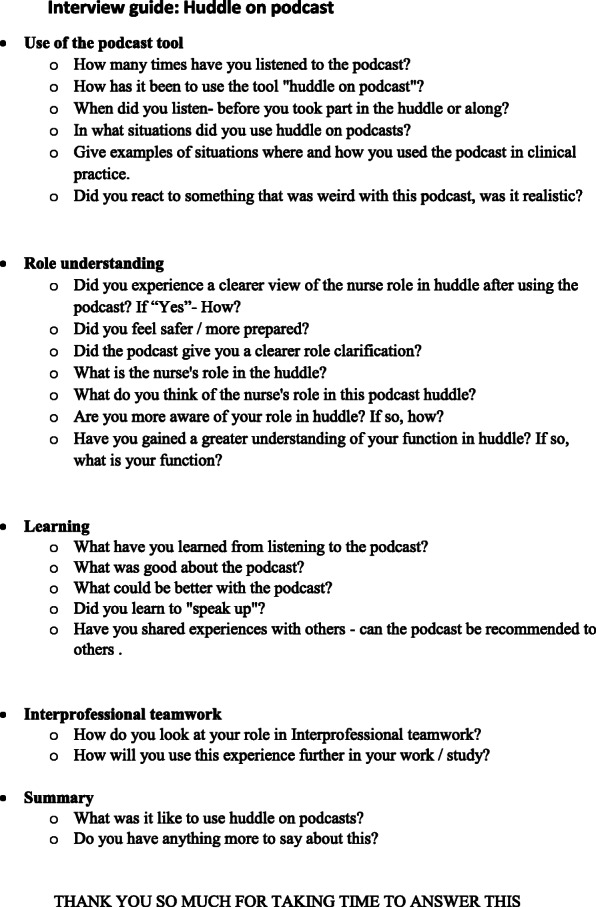
Interview guide: Huddle on podcast

This qualitative and exploratory pilot study used focus group interviews [24]. A semi-structured interview guide (see: Fig. 1) was developed covering the following topics: understanding one’s own professional role, understanding interprofessional communication and teamwork, usability of the learning tool and learning experience.

### The podcast

The development of the huddle-focused podcast was inspired by Wenger’s theory, communities of practice [25], which focuses on practice as the core arena in which a student-group as a community develops, shares, and maintains its learning. Furthermore, the communication structure adopted in the podcasts followed the Situation, Background, Assessment, and Recommendation (SBAR) format. SBAR is a situational briefing tool designed to function as a checklist, as well as a means of structuring a team’s information exchange, as recommended by the World Health Organization [4, 26].

The patient cases were developed in cooperation with nurses and physicians at a nephrology medical ward in a Norwegian university hospital, and teachers and researchers from the university. The healthcare professionals provided examples of relevant and realistic patient cases. The researchers developed podcast manuscripts consisting of spoken scripts that replicated the dialogue between a nurse and a physician participating in a huddle. Before recording the podcasts, the manuscripts were qualitatively validated for relevance to the healthcare professionals and for reliability in a healthcare setting. Each podcast lasts approximately four minutes.

With support from the university’s communication and technical department, the authors transferred the patient cases to the podcast by recording the voices of nurses and physicians. The university department also helped with uploading and distributing the podcast.

The three cases used in the podcast covered the discharge of an older patient, the care of a middle-aged patient with an acute pyelitis, and the treatment of an older patient with chronic renal failure receiving hemodialysis.

### The study context

The hospital (here: Medical department) is, together with the university, responsible for learning outcomes in practice, there is a binding collaboration between the university and the hospital in the facilitation and implementation of practice studies for nursing students.

The nursing students were in their third year, and at this stage their focus for learning is holistic and collaboration nursing. Before the students began their clinical placement period in the medical ward, they were informed both orally and in writing about the podcast project and its focus on huddle case scenarios. The students gained access to the podcasts through links sent to their email address and the clinical teacher encouraged the students to use the podcast before and during their practical period. Listening to the podcast was not part of the graded work for the students and they were not required to listen to it.

### Participants

Twenty nursing students, all of whom had their clinical placement on the medical ward, and were in either their fifth or sixth semester, were invited to participate in this pilot study. Of these, 11 consented to participate (six in their sixth, semester and five in their fifth semester, 10 females and one male, and a mean age of 26 years). Each focus group comprised five, four, or two participants. For practical reasons, the interviews were held at the end of the workday. The students that had been on the same shift, were therefore participating in the same interview. An advantage of this was that they knew each other and spoke easy together.

### Data collection

Three focus group interviews; one with each of the three groups (each group in one of three different practice periods) were conducted with the nursing students by the first and last authors. Both are experienced qualitative researchers. We were aware of the difficult ethical issues introduced by the inherently blurred boundaries in pedagogical inquiries between the roles of the researchers and the participants [29]. The fact that the researchers in this study were working at the educational institution could cause a risk of influence on participants’ answers. This risk was reduced by not allowing researchers to be both supervisors and interviewers of the participants. Furthermore, the interviews were held after students’ clinical placement assessments, so it was clear that nothing the students said in their interviews would influence that evaluation.

The interviews took place in the students’ clinical placement location in the hospital. The interviews lasted an average of 30 min. The focus group interviews were managed by two researchers to make reliable observations and avoid “moderator dominance” and be aware of that the students’ voices were given equal opportunity to be heard. Audiotaped recordings of each interview where transcribed and analyzed.

### Ethical consideration

The study was approved by the Norwegian Centre for Research Data (116097). Participation required informed, voluntary, written consent, and students were informed about the right to withdraw from the study at any point. The students were assured that neither participation nor non-participation would affect other aspects of their placement period or give them any advantages or disadvantages along their educational pathway.

### Analysis

A qualitative content analysis of the transcribed interviews was conducted to capture textual content related to the research aims [27]. The material was subsequently combined into a single text that was subjected to the researchers’ scrutiny and qualitative content analysis. “Meaning units” (i.e., groups of words or phrases reflecting similar content and context) were identified, condensed and coded. The condensed data were organized into sub-categories and aggregated into categories labeled with different colors that reflected the content of nursing students’ experiences with the huddle-related podcasts. Following Polit and Beck [24] and Graneheim and Lundman [27], content analysis was adopted to enrich our reflections on the data and our interpretations of them. All three authors read the transcripts through independently to obtain a general impression of the dataset. They met several times to discuss the coding process and to finalize the categories. Saturation was discussed during analysis process. We concluded that saturation was reached when the data did not reveal any new categories that were considered relevant for the research question [24, 27].

## Results

The analysis identified four categories that resonated across all participants in the focus group interviews: 1. understanding one’s own role in the huddle; 2. encouraged to speak up; 3. a flexible learning tool; and 4. authentic but not always realistic (see Table 1: Categories and Sub-categories).

**Table 1:** Categories and Sub-categories. The analysis identified four categories that resonated across all participants in the focus group interviews: 1. understanding one’s own role in the huddle; 2. encouraged to speak up; 3. a flexible learning tool; and 4. authentic but not always realistic.

Categories	Sub-categories
Understanding one’s own role in the huddle	• Clearer picture of how to communicate and cooperate • Insight into what a huddle includes • Proud of own profession and role • Own role is more clear • Collaboration with physicians • Voice as the patient’s advocate
Encouraged to speak up	• Reduce anxiety and diminish stress • Insecurity about expectation • Assumed the physicians know best • Speak with greater confidence
A flexible learning tool	• Useful and relevant • Easy to use • Listen to everywhere • Listened to before and after the huddles • Listened to before and during the clinical placement • Used as reassurance
Authentic but not always realistic	• Ideal versus real life- more in-depth than huddles in practice • Beneficial to outline the patient cases • Starting with ice breaker between the professional groups

### Understanding one’s own role in the huddle

Gaining a better understanding of their own role in the huddle seemed to be an important benefit of listening to the podcasts. In most cases, students struggled to get a clear picture of what their roles were when cooperating with the physicians in the huddle. They described being unsure of how they should contribute during the huddle sessions. For instance, what type and amount of patient information should they present to the physicians and to what degree should they express their own reflections about the patients’ treatments? These sentiments are illuminated in the following quote:

*.. it is a bit undefined what is really my role here. We will talk together, but about what? How much should I say? (FG1).*

When listening to the huddles presented on the podcast, the students seemed to arrive at a clearer picture of how to cooperate and communicate with the physicians. The podcasts gave the students more insight into what a huddle was, and what topics to include, both of which increased their understanding of their own role in the huddle. The students explained that they were no longer completely lost about what to say or discuss with the physicians when they entered the huddle and attributed this newfound confidence and knowledge to the podcasts. For example, some students realized that they had a voice as the patient’s advocate, and they also felt a sense of pride in their own profession and role. Students believed that a nurse’s role in the huddle was clearer because that role had been delineated in earlier practice placements. This is illuminated in the following quote:

*… the role of the nurse in the huddle is important; by reporting everything to the physician, that allows the physician to make an assessment. In addition, the patient must have the feeling of being taken care of. (FG1).*

Cooperation between nurses and physicians in a huddle was highlighted as important for patient care. Students described the importance of proposing measures or activities for the patients, and of coming up with other suggestions, to which the physicians responded positively and thus believed that they had been heard. Because, as they explained: “*the physicians are not with the patient all the time, while nurses spend more time at the patient’s bedside; we are there much more.*” *(FG2).*

The students did not talk only about what they had learned about their own roles as nurses, but also of the important role of their collaborating physician in the huddles. Thus, they indicated that also providing medical students with the podcasts would be valuable, because both groups of students would then share equal knowledge about huddles.

### Encouraged to speak up

The nursing students noted that listening to the podcast reduced their anxiety and made it easier for them to speak out more confidently in the huddle meeting.

The huddle seemed to represent something “unknown” to the students, unlike many other practical skills and procedures they have learned about in the study program. In the huddle meeting they said they admitted that it was something they hadn’t really grasped during their previous nurse education. They described their anxiety when they knew that taking part in the huddle would be a part of their learning activity within the next days of their clinical placement. However, when the podcast gave them specific examples of what a huddle could look like, their stress seemed to diminish because then they knew what to expect. Some students expressed it like this:

*Then it was very scary to join it. And I had no idea what the huddle was until I heard the podcast, since I have not been to a huddle before. When I heard everything you guys said I thought, oh yeah, that’s how it works. So that made me feel safer. (FG1).*

*A little scary at first, but when you heard that recording then I got a little more relaxed and thought I should be able to do this. (FG2).*

This anxiety seemed closely linked to insecurity about what was expected from them during the huddle. Clearly, the students have a deep respect for the physicians’ profession and knowledge and tried to “read between the lines” when it came to how each physician wanted the huddle to be conducted. The students described trying to adjust to different physicians:

*Yes, it’s always a little difficult because you do not quite know how the physicians like it in the ward. So often you say either too much or too little. Maybe preferably, often, too little. (FG3).*

*It depends on which physician you go with, how comfortable you feel, whether it is, free of discussion or if it is a chief physician who has the attitude like “That’s how it will be.” (FG2).*

One student shared that before she had listened to the podcasts that she “felt like a mouse” and assumed that the physician knew best. She was aware of her own shortcomings and described the huddle as a hierarchy in which the physicians enjoyed a higher professional status than nurses.

However, by listening to the podcasts, participants felt encouraged to speak with greater confidence. They talked about physicians who “got hung up on” their perspectives in the treatment of the patients and stated that the podcasts encouraged them express their own nursing perspectives:

*Maybe the podcast could also help you as a nursing student to dare to take a little more space in the huddle.* (FG1).

### A flexible learning tool

Another issue was the flexibility and ease of learning afforded by the podcasts. Students reported listening to the podcasts in the car or on the bus on the way to or from their clinical practice.

The podcast was described as a practical and flexible tool that was easy to use and could be listened to everywhere.

The findings also showed that the students found the podcast huddle cases useful and relevant. They insisted that it helped them prepare for their huddles and to recall previous huddle experiences from their second-year hospital clinical placements period. Students said they had listened to the different cases presented on the podcasts before and during the clinical placements period. They also reported that the podcasts gave them an overview of what a huddle should or could be. As one student said:

*I think it gave a good insight; it seems simple. An overview, in a way. So, you are not completely lost when you enter the huddle. (FG2).*

The students stated that it was very convenient to acquire knowledge about huddles before they started in the hospital clinical placement in their second year. One student said:

*I remember the second year, it was very uncomfortable to have the huddle, but now in the third year with the podcasts it is rather natural. (FGI).*

Additionally, students agreed that even though they had experienced huddles before, it was good to use the podcast for reassurance:

*But I think it was very nice to have … when I heard it for the first time, it was a bit like; this is going well, because I had dreaded the huddles, because it was long since we have had one. We do not have it in primary care as I was in last clinical placement. (FG3).*

To reinforce their knowledge of the huddle, one student suggested developing a type of huddle that covers important points to support the daily huddles. The students also mentioned that the huddle-focused podcasts could be useful for the physicians, enhancing their awareness of nurses’ and physicians’ roles during a huddle and, in certain situations, paint “a broader picture” of the patient. One student said:

*The physician was reminded that something was caught there that would not have been caught if we had not gone and if the nurse had not been given room to talk. (FGI)*

### Authentic but not always realistic

The students stated that the podcasts were not always a realistic reflection of their daily work; that is, not every detail described in the podcast cases aligned with their own experience of the huddles in their clinical practice. For instance, the podcast cases were more in-depth than their experiences with daily huddles in practice. On the ward, students experienced the huddle as being more “to the point.” They described their real-life huddles as shorter and often affected by the fact that the physicians and nurses already knew the patients and that the physicians were busy, which sometimes meant there was very little, if any, time for a huddle. However, participants conceded that it could be beneficial to outline the patient cases in a huddle as illustrated in the podcast. Furthermore, starting the podcast with an icebreaker between the professional groups could reduce students’ anxiety around the pre-visit, as expressed in the following quote:

*And just the fact that for example, talk about everyday things in the podcast makes you become a little more like that; actually, not that scary. (FGI).*

## Discussion

The aim of this study was to explore nursing students’ experiences with a learning tool focusing on interprofessional teamwork in the huddle. In general, our findings indicate that the podcast, which requires few resources, has considerable educational potential. The huddle-focused learning tool offered even greater benefits and functionality than we had expected. This provides justification for further development of the podcast.

An important finding is that the podcast helped students to better understand their own roles in their daily huddles and thereby built their confidence. Role-related stereotypes seem to persist in nursing students’ perceptions, manifesting the view that physicians are more powerful than nurses. These results are in line with other studies of the hierarchy, roles, and stereotypes that still exist in collaborations between nurses and physicians, and in the dominance of medical power [11, 12]. It was surprising to see how “small” the nursing students viewed themselves, how inferior they felt in the huddle setting. The nursing role seems immature to the third-year students. These findings indicate the lack of focus on huddle teaching program and interprofessional training in the nursing program.

Our findings indicate that the students felt encouraged to speak up in their huddle meetings after listening to the podcasts. Communication is a core issue in interprofessional teamwork [4], as evidenced by extensive research showing the steady rate at which communication errors are identified as the cause of unwanted incidents in health services [6, 7, 9]. If podcasts can help empower nursing students to speak up in the huddle, they might be encouraged to do so also in other interprofessional collaboration contexts. This might prevent communication errors and unwanted incidents in patient care.

As this is a pilot study, it is important to reflect on how the study findings can facilitate the improvement of the podcast. We have argued that it was important that the podcast was developed in cooperation with clinical health care professionals. Through this partnership between the educational institution and a clinical practice, we aimed to jointly develop quality assured exemplars of interprofessional teamwork and communication for huddles that can be considered as a “gold standard” for interprofessional activity and that nursing students will perceive as relevant to their practice studies. However, the students found that the cases were not always realistic indicating a gap between the educational institution and the hospital. Based on this, it might be useful to involve clinicians even more.

Furthermore, the involvement of nursing students might have increased the relevance of the cases. Co-creation is highlighted as a social process in which the creators and the users of knowledge together determine the tool’s appropriateness in clinical studies [28]. Therefore, increasing the collaboration and involvement of clinicians and nursing students will be a goal for future educational podcasts.

With respect to usability, students considered the podcast easy to use and did not report any technical problems. Participants used the podcast as part of their huddle preparation (e.g., to reinforce their knowledge), and found that they could easily listen to it anywhere and at any time, whether at work, at home, or in between. These findings are in line with Shunk et al. [17]. and O’Connor et al. [20], all of whom noted the potential of podcasts as a learning tool. Extending this idea, podcasts have potential in other clinical educational settings such as patient education sessions, nurse reporting, and overlap discussions.

### Limitations

A limitation in the study is the small number of participants. However, as this is a pilot study, we argue that the amount of data is sufficient to gain valuable insights that can facilitate choices in the refinement of the huddle-focused learning tool.

## Conclusions and implications

Our findings indicate that the students perceived that the podcasts helped them to get insight in how to engage in interprofessional teamwork. As a direct result of listening to the podcast, the students gained a better understanding of their own role in their daily huddle meetings, and also felt encouraged to speak up in those meetings.

A podcast as a “learning tool” is easy to use and can be readily adapted to other educational settings in nursing programs. Possible relevant subjects could be communication with patient and next of kin in different situations such as information at discharge or preparation for surgery. Other option could be to demonstrate oral nurse reports.

Overall, the findings from this pilot study can provide nursing programs with important knowledge and insights about how nursing students can use educational podcasts to learn role clarity and encourage equal interprofessional communication and collaboration. The huddle in nursing education offers tremendous potential as content for a learning tool, so podcast development should include input both from medical students and from a variety of realistic patient cases.

## Data Availability

Data used in the present study are available from the corresponding author on request.

## Abbreviations

AHRQ: Agency for Healthcare Research and Quality
SBAR: Situation, Background, Assessment, and Recommendation
WHO: World Health Organization

## Availability of data and materials

The data used in the study are available from the corresponding author on request.
